# Supplementary material for: Fungistatic Action of N-Acetylcysteine on Candida albicans Biofilms and Its Interaction with Antifungal Agents
Source: Microorganisms. 2020 Jun 30;8(7):980. doi: 10.3390/microorganisms8070980 (PMC7409114; doi:10.3390/microorganisms8070980)
Supplement: Supplementary file 1 [file microorganisms-08-00980-s001.pdf]

Supplementary Materials

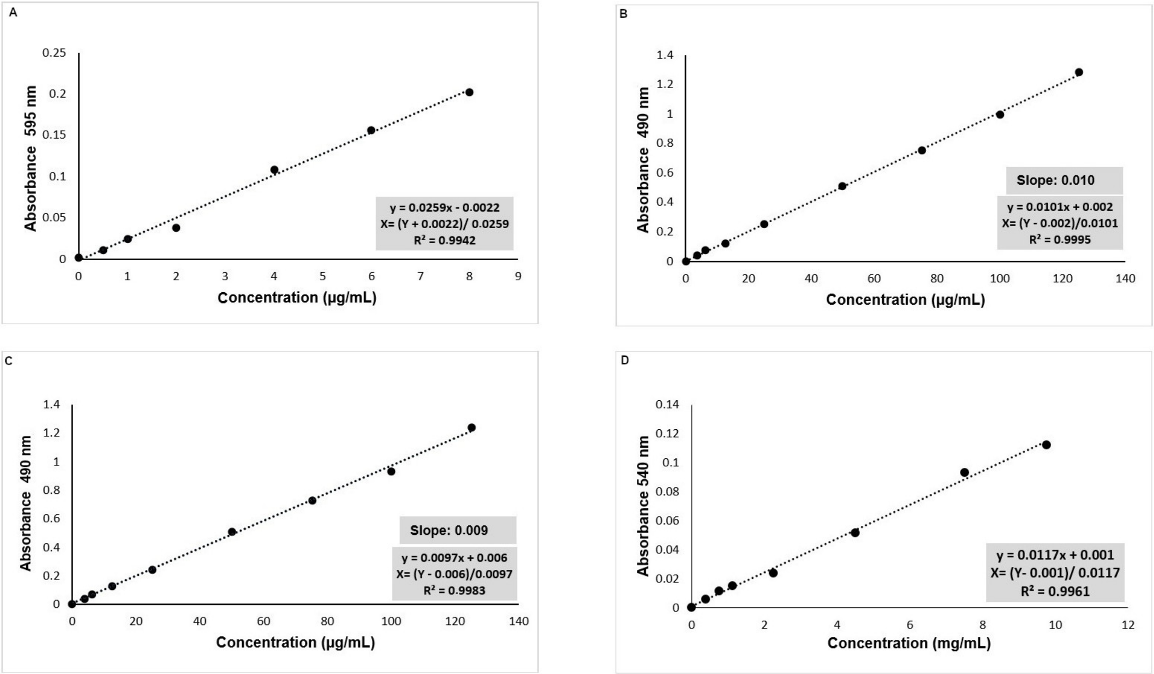

Figure S1: Standard curve of soluble proteins (A), WSP (B), ASP (C) and lipids (D).

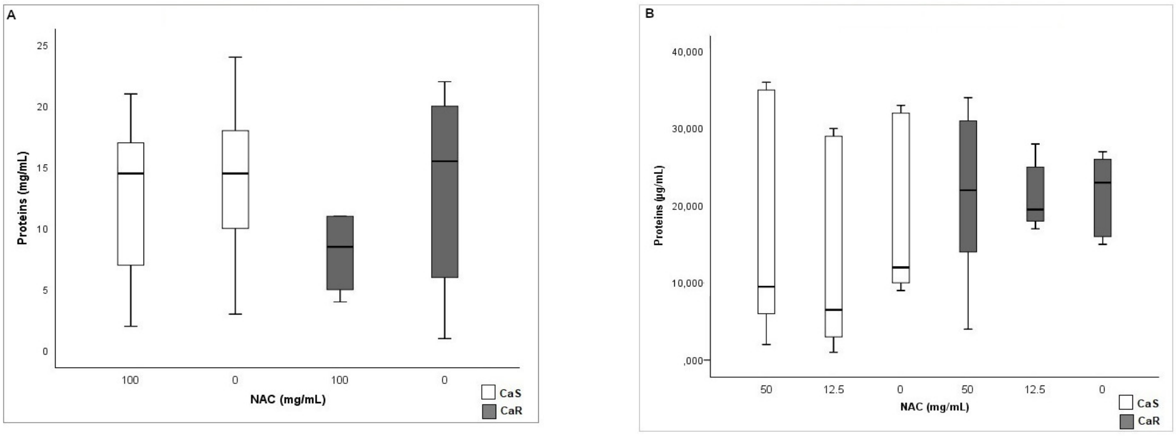

Figure S2: Insoluble protein values obtained for CaS and CaR for pre-formed biofilms (A) and biofilm formation (B). Dash: median; top box: 3rd quartile; bottom box: 1st quartile; whiskers: maximum and minimum values.

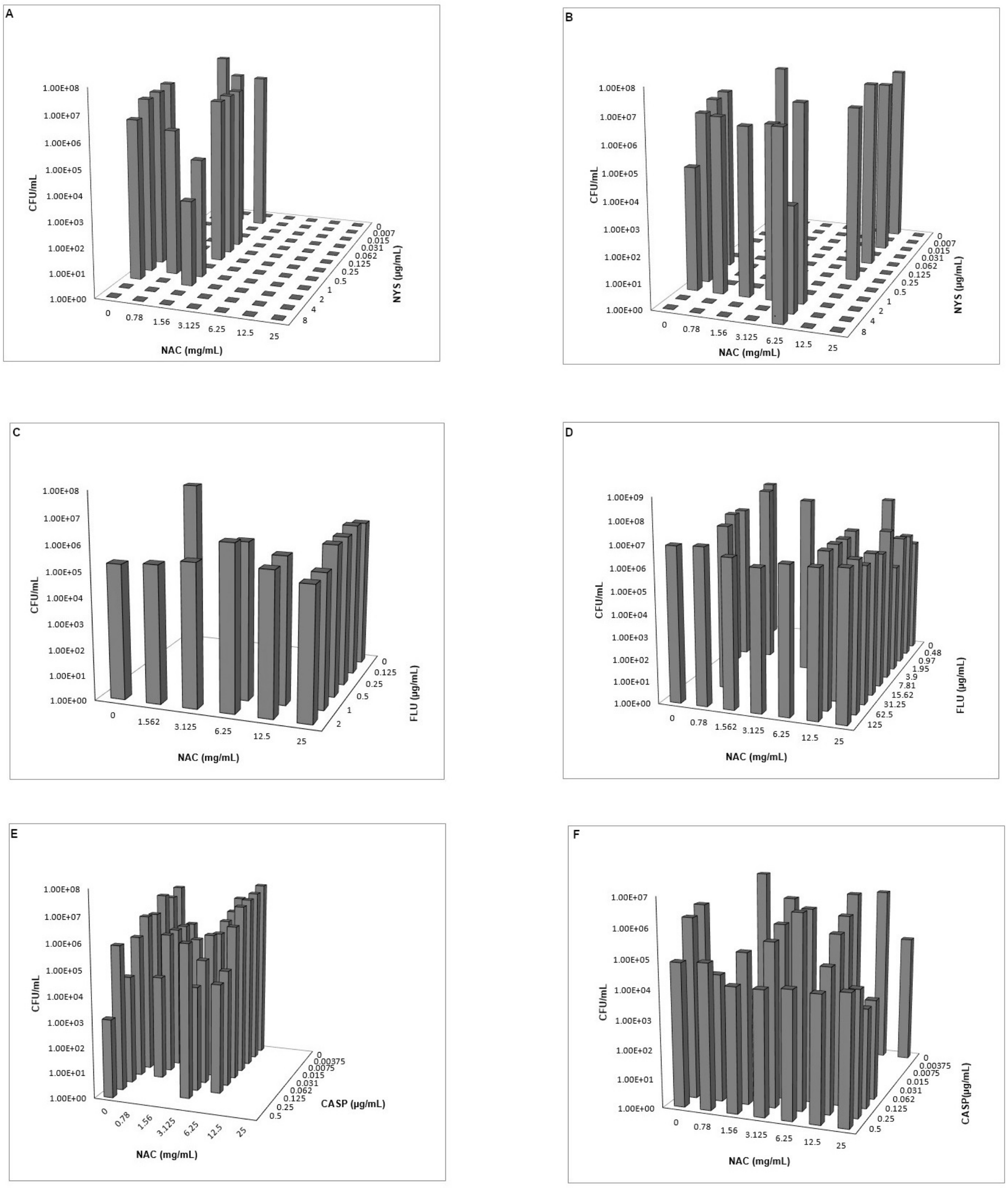

Figure S3: Mean values of viability of planktonic cultures obtained from the interaction of NAC with Nystatin for CaS (A) and CaR (B); NAC with Fluconazole for CaS (C) and CaR (D); and NAC with Caspofungin for CaS (E) and CaR (F).
